# Associations of Vision, Hearing, and Dual Sensory Impairments With Functional Outcomes: A Nationwide Analysis

**DOI:** 10.7759/cureus.116011

**Published:** 2026-09-09

**Authors:** Sarah M Kaiwa, Mohammad Abdalhalem, Khalid M Alqahtani, Remaz A Alharbi, Atheer K Alolayt, Shahad A Alhaddad, Wiam E Alghafli, Ziyad E Alsadi, Ahmed O Hafeezallah, Khalid M Aloufi

**Affiliations:** 1 College of Medicine, Taibah University, Medina, SAU; 2 Department of Otolaryngology-Head and Neck Surgery, Taibah University, Medina, SAU; 3 College of Medicine, King Khalid University, Abha, SAU; 4 College of Medicine, Imam Abdulrahman Bin Faisal University, Dammam, SAU; 5 College of Medicine, Vision College, Riyadh, SAU; 6 College of Medicine, University of Jeddah, Jeddah, SAU

**Keywords:** concurrent sensory impairment, dual sensory impairment, functioning, hearing impairment, hearing loss, quality of life, vision impairment, vision loss

## Abstract

Introduction

The co-occurrence of hearing and vision impairment, known as dual sensory impairment, is thought to impose a larger functional burden than either impairment alone. The purpose of this study was to identify characteristics that are independently related to dual sensory impairment and investigate its relationship with functional limitations across five domains of daily living in comparison with isolated sensory impairment.

Methods

This retrospective, cross-sectional study utilized data from the 2023 National Health Interview Survey (NHIS), using sampling weights, strata, and clusters to produce nationally representative estimates. A population of 29,522 adults was included in the analysis. Vision and hearing impairments were identified through self-reported difficulties, and individuals with both were classified as having dual sensory impairment. Five domains of functional impairment were evaluated: communication, social participation, independent errand performance, ambulation, and self-care. Multivariable logistic regression, while controlling for demographic and clinical covariates, was conducted to identify factors associated with dual sensory impairment and to assess the relationship between sensory impairment status and functional limitation, with results presented as adjusted odds ratios (aORs) and 95% confidence intervals (CIs).

Results

Of the 29,522 participants, 1,858 (5.6%) exhibited dual sensory impairment. This group was significantly older and had higher rates of obesity and smoking, as well as poorer self-rated health and lower life satisfaction, compared to individuals without sensory impairment. Dual sensory impairment was independently associated with older age, smoking history, hypertension, stroke, coronary artery disease (CAD), cancer, chronic obstructive pulmonary disease (COPD), dementia, anxiety, and depression (all p < 0.05); the association with diabetes mellitus was no longer statistically significant after adjustment. Throughout all five functional areas, dual sensory impairment exhibited the strongest association with impairment in comparison to solely visual or hearing impairment, encompassing ambulation (aOR: 3.87), self-care (aOR: 3.55), communication (aOR: 6.03), independent errands (aOR: 3.81), and social participation (aOR: 4.08) (all p < 0.001).

Conclusion

Dual sensory impairment is prevalent among a significant number of adults in the United States and is associated with considerably higher odds of functional limitation across multiple domains compared with isolated visual or hearing impairment.

## Introduction

Vision and hearing are the two senses by which people interact with their surroundings and communicate with others and, depending on them, carry out their daily activities. The impairment of vision and hearing is related to aging, which is highly prevalent worldwide. The World Health Organization indicates that more than 430 million individuals worldwide suffer from hearing loss, and it is expected to exceed 700 million by 2050 [1]. Also, global indications from the 2020 Global Burden of Disease Study show that individuals who suffer from moderate-to-severe vision impairment are around 295 million, and 43 million individuals are classified as blind [2]. As aging is a continuous process, these impairments are expected to affect more people, which will increase the burden on both healthcare systems and individuals [3]. Furthermore, dual sensory impairment rates were the highest among individuals older than 79 years of age, according to a nationwide analysis [4].

Although the effects of visual and hearing impairment individually on functional capacity, cognitive performance, and the quality of life have been extensively documented, increasing evidence suggests that the presence of both impairments, termed dual sensory impairment, constitutes a distinct and more severe condition than either impairment alone [5]. Since vision and hearing work together in facilitating communication, spatial orientation, and the awareness of the surrounding environment, the simultaneous impairment of both senses may intensify functional limitations beyond the effects of either deficit alone, thereby increasing the risk of loss of independence. Among older adults, dual sensory impairment has also been associated with higher rates of depression, cognitive decline, and reduced well-being compared with isolated sensory impairment [6].

Much of the existing literature has examined the recognized interaction between hearing and vision impairment but has focused primarily on regional cohorts or a single functional outcome. The lack of sufficient national representation limits the generalizability of these findings to the wider population. As the number of older adults in the United States is expected to increase from 58 million in 2022 to 82 million in 2050, nationally representative data are needed to describe the burden of dual sensory impairment and its functional impact [7]. Surveillance systems such as the National Health Interview Survey (NHIS) provide an opportunity to examine this relationship using a multistage sampling strategy at the national level while enabling the simultaneous assessment of multiple domains of daily functioning [8]. This approach remains underutilized in the dual sensory impairment literature, although there is evidence that greater impairment severity is associated with progressively poorer quality of life [9]. This study had two specific objectives: (1) to identify the demographic and clinical characteristics independently associated with dual sensory impairment, using individuals without dual sensory impairment as the comparison group, and (2) to evaluate the independent associations of isolated visual impairment, isolated hearing impairment, and dual sensory impairment, each compared to a reference group of individuals with no sensory impairment, with functional limitation across five pre-specified domains of daily living: ambulation, communication, self-care, independent performance of errands, and social participation.

## Materials and methods

Study design and population

This is a retrospective analysis of cross-sectionally gathered, nationally representative data from the 2023 NHIS. Data in the NHIS database are gathered from adults in the United States using a multistage sampling technique [8].

We gathered data from the 2023 survey for the purpose of this research. Survey weighting, clustering, and stratification were utilized to establish proper national representativeness. We included all adults from the 2023 NHIS survey (n = 29,522). In this study, informed consent was established in adherence with Beth Israel Deaconess Medical Center policy. The National Center for Health Statistics approved the data reported by the NHIS to ensure the privacy, data quality, and integrity of scientific material.

We have included all non-institutionalized individuals who are 18 years of age or older who completed the sample adult questionnaire of the 2023 NHIS. Those with missing, refused, or “don’t know” responses to the questions assessing visual or hearing functions, questions related to the outcome (functioning), or questions related to covariates such as comorbidities and sociodemographic factors were excluded.

Dependent variable (outcome)

In our study, the primary outcome of interest was functional impairment. Five major domains of functioning were particularly assessed, which were ambulation, communication, self-care, performing errands independently, and participating in social activities. Every domain of these five domains was assessed by a single question; for ambulation, participants were asked, “Do you have difficulty walking or climbing steps? Would you say no difficulty, some difficulty, a lot of difficulty, or you cannot do this at all?” Communication was assessed with the following question: “Using your usual language, do you have difficulty communicating, for example, understanding or being understood?” Self-care was evaluated by asking, “Do you have difficulty with self-care, such as washing all over or dressing? Would you say no difficulty, some difficulty, a lot of difficulty, or you cannot do this at all?” Performing errands independently was assessed with the following question: “Because of a physical, mental, or emotional condition, do you have difficulty doing errands alone, such as visiting a doctor’s office or shopping?” Participating in social activities was evaluated by asking, “Because of a physical, mental, or emotional condition, do you have difficulty participating in social activities such as visiting friends, attending clubs and meetings, or going to parties?” Those who answered with “some difficulty,” “a lot of difficulty,” or “cannot do at all” were considered to have difficulty in the respective domain.

Independent variables

Our exposure of interest in this analysis was dual sensory impairment, in the form of concurrent hearing and visual impairment. Vision was assessed by asking the participants, “Do you have difficulty seeing, even when wearing glasses or contact lenses? Would you say no difficulty, some difficulty, a lot of difficulty, or you cannot do this at all?” Hearing function was evaluated with the following question: “Do you have difficulty hearing, even when using your hearing aid(s)? Would you say no difficulty, some difficulty, a lot of difficulty, or you cannot do this at all?” Those who answered with “cannot do at all,” “a lot of difficulty,” or “some difficulty” were considered to have hearing/visual impairment. Those who were considered to have impairment in both questions were considered to be dual sensory impaired. Several demographic and clinical covariates were taken into account, such as age, gender, body mass index (BMI), self-reported general health status, self-reported life satisfaction, history of smoking, diabetes mellitus, hypertension, stroke, coronary artery disease (CAD), cancer, chronic obstructive pulmonary disease (COPD), dementia, anxiety (positive Generalized Anxiety Disorder-2 {GAD-2} screener) [10], and depression (positive Patient Health Questionnaire-2 {PHQ-2} screener) [11].

Statistical analysis

Clinical and demographic characteristics were summarized using appropriate descriptive statistics. Categorical variables were presented as frequencies and percentages and compared between groups using chi-square tests. Age was the only continuous variable in this analysis, and the Shapiro-Wilk test was used to assess normality; it was found to be normally distributed and is reported as mean ± standard error (SE), compared between groups using an independent-samples t-test. All remaining variables were categorical and are reported as frequencies with survey-weighted percentages, compared using design-adjusted chi-square tests.

To account for the complex survey design, analyses incorporated the sampling weights, strata, and primary sampling units (clusters) to obtain nationally representative estimates and appropriate standard errors and confidence intervals (CIs). Multivariable logistic regression models were used to examine the independent associations between selected predictors and binary outcomes, with results reported as adjusted odds ratios (aORs) with 95% confidence intervals (CIs). Statistical significance was defined as p < 0.05. Statistical analyses were performed using Stata 15.1 (StataCorp LLC, College Station, TX), while Python 3.8 (Python Software Foundation, Fredericksburg, VA) and Matplotlib were used for data visualization.

In regard to each of the regression models, individuals who did not have either a visual impairment or an auditory impairment served as the reference category for the sensory impairment exposure. The three types of sensory impairments (hearing impairment only, visual impairment only, and dual sensory impairments) were defined as mutually exclusive and were compared to the reference category. Each of the descriptive analyses and the regression models included the NHIS sampling weights, strata, and primary sampling units in their entirety. No analysis was performed without accounting for the complex survey design. Individuals with missing, refused, or “don’t know” answers to any exposure variable, outcome variable, or covariate variable were eliminated from all analyses before they began (as mentioned earlier).

## Results

Participant characteristics

Twenty-nine thousand five hundred twenty-two non-institutionalized individuals in the United States from the 2023 NHIS database were included in this study. Twenty-seven thousand six hundred sixty-four (94.4%) were individuals with non-dual impairment, and 1,858 (5.6%) had dual sensory impairment.

Individuals suffering from dual sensory impairment were significantly older (mean age: 58.69 ± 0.55) compared to those without impairment (47.39 ± 0.16, p < 0.001). Regarding gender distribution, there was no significant difference between the groups, with women constituting about 51.9% of those with dual impairment and 51.2% of non-impaired individuals (p = 0.57).

As for body mass index, there was a significant difference between the groups. Obesity was found to be more common among individuals with dual sensory impairment compared to those without impairment (38.0% versus 32.9%, p < 0.001). Similarly, a healthy weight was less common among those with dual sensory impairment in comparison to individuals without impairment (26.5% versus 30.2%, p < 0.001).

Moreover, participants who had dual sensory impairment were more likely to report poorer general health when compared to those without impairment (13.4% versus 2.8%, p < 0.001). Out of those with dual impairment, only 6.5% reported their health to be excellent, compared to 23.1% of individuals without impairment (p < 0.001).

Participants with dual sensory impairment were also less likely to report life satisfaction as very satisfied (26.2% versus 45.3%) and more likely to report life satisfaction as dissatisfied (10.3% versus 3.1%) or very dissatisfied (2.0% versus 0.7%) (all p < 0.001).

Regarding the clinical characteristics of participants, smoking was significantly more common among those with dual sensory impairment (47.8% versus 31.2%, p < 0.001). Likewise, comorbid chronic conditions were consistently more common in individuals with dual impairment when compared to participants with non-dual impairment, as they had higher rates of diabetes mellitus (18.9% versus 9.4%), hypertension (52.8% versus 31.2%), stroke (9.4% versus 2.7%), coronary artery disease (13.5% versus 4.5%), cancer (19.4% versus 9.2%), chronic obstructive pulmonary disease (14.7% versus 3.8%), dementia (5.7% versus 0.9%), anxiety (35.9% versus 17.6%), and depression (40.0% versus 17.7%) (all p < 0.001) (Table 1).

**Table 1:** Baseline characteristics of participants with dual sensory impairment and with no dual sensory impairment. Values are presented as unweighted frequencies (n) with survey-weighted percentages (%) for categorical variables and survey-weighted mean ± standard error (SE) for age. Because percentages are derived from NHIS sampling weights, strata, and cluster design, weighted percentages do not correspond directly to the unweighted n/N ratio; this is expected and reflects nationally representative estimation. All analyses accounted for the complex survey design, including sampling weights, stratification, and clustering by primary sampling units. We compared categorical variables using design-adjusted chi-square tests and age using a survey-adjusted independent-samples t-test. We considered a two-sided p-value of <0.05 to be statistically significant. CAD, coronary artery disease; COPD, chronic obstructive pulmonary disease; BMI, body mass index; NHIS, National Health Interview Survey

		Total	No dual sensory impairment		Dual sensory impairment	Statistic value	P-value
Number (%)		29,522	27,664 (94.4)		1,858 (5.6)		
Gender (female, n {%})		16,065 (51.2)	15,060 (51.2)		1,005 (51.9)	Chi-square = 0.38	0.57
Age (years, mean ± SE)		53.04 ± 0.28	47.39 ± 0.16		58.69 ± 0.55	T value = -19.96	<0.001
BMI category							
Underweight (yes, n {%})		458 (1.6)	420 (1.6)		38 (1.9)	Chi-square = 22.2	<0.001
Healthy weight (yes, n {%})		8,871 (30.0)	8,351 (30.2)		520 (26.5)		
Overweight (yes, n {%})		10,568 (35.3)	9,948 (35.4)		620 (33.6)		
Obese (yes, n {%})		9,625 (33.1)	8,945 (32.9)		680 (38)		
	General health status						
Excellent (yes, n {%})		6,046 (22.1)	5,936 (23.1)		110 (6.5)	Chi-square = 1,249.02	<0.001
Very good (yes, n {%})		9,900 (33.5)	9,526 (34.3)		374 (19.8)		
Good (yes, n {%})		8,742 (29.2)	8,153 (29.1)		589 (31.5)		
Fair (yes, n {%})		3,723 (11.8)	3,189 (10.8)		534 (28.8)		
Poor (yes, n {%})		1,111 (3.4)	860 (2.8)		251 (13.4)		
	Life satisfaction						
Very satisfied (yes, n {%})		12,882 (44.3)	12,393 (45.3)		489 (26.2)	Chi-square = 428.01	<0.001
Satisfied (yes, n {%})		15,316 (51.1)	14,178 (50.9)		1,138 (61.5)		
Dissatisfied (yes, n {%})		1,079 (3.5)	892 (3.1)		187 (10.3)		
Very dissatisfied (yes, n {%})		245 (0.7)	201 (0.7)		44 (2.0)		
History of smoking (yes, n {%})		10,373 (32.1)	9,461 (31.2)		912 (47.8)	Chi-square = 196.05	<0.001
Diabetes mellitus (yes, n {%})		3,327 (9.9)	2,935 (9.4)		392 (18.9)	Chi-square = 160.34	<0.001
Hypertension (yes, n {%})		11,145 (32.4)	10,061 (31.2)		1,084 (52.8)	Chi-square = 330.86	<0.001
Stroke (yes, n {%})		1,171 (3.1)	964 (2.7)		207 (9.4)	Chi-square = 232.21	<0.001
CAD (yes, n {%})		1,925 (5.0)	1,622 (4.5)		303 (13.5)	Chi-square = 262.29	<0.001
Cancer (yes, n {%})		3,800 (9.8)	3,363 (9.2)		437 (19.4)	Chi-square = 187.05	<0.001
COPD (yes, n {%})		1,654 (4.4)	1,346 (3.8)		308 (14.7)	Chi-square = 440.23	<0.001
Dementia (yes, n {%})		396 (1.1)	288 (0.9)		108 (5.7)	Chi-square = 321.34	<0.001
Anxiety (yes, n {%})		5,487 (18.6)	4,860 (17.6)		627 (35.9)	Chi-square = 343.48	<0.001
Depression (yes, n {%})		5,814 (19.0)	5,089 (17.7)		725 (40.0)	Chi-square = 500.85	<0.001

Factors independently associated with dual sensory impairment

Multivariate regression analysis showed that greater odds of dual sensory impairment were present with increasing age (aOR: 1.03; p < 0.001). Compared with healthy weight, the difference with other BMI categories (underweight, overweight, and obese) was not found to be statistically significant.

Individuals with a history of smoking cigarettes had substantially higher odds of dual sensory impairment compared to those with a negative smoking history (aOR: 1.32; p < 0.001).

Most of the comorbid conditions included in our analysis were found to be independently associated with dual sensory impairment, namely, hypertension (aOR: 1.21; p = 0.004), stroke (aOR: 1.48; p < 0.001), coronary artery disease (aOR: 1.34; p = 0.002), cancer (aOR: 1.20; p = 0.011), COPD (aOR: 1.66; p < 0.001), dementia (aOR: 2.04; p < 0.001), anxiety (aOR: 1.69; p < 0.001), and depression (aOR: 2.10; p < 0.001), whereas diabetes mellitus was not found to be independently associated with dual sensory impairment after adjustment for confounding factors (aOR 1.11; p = 0.20) (Table 2).

**Table 2:** Multivariate logistic regression analysis of factors associated with dual sensory impairment. Results are presented as adjusted odds ratios (aORs) with 95% confidence intervals (CIs) and corresponding p-values. The regression model accounted for the complex survey design by incorporating sampling weights, stratification, and clustering by primary sampling units. All variables shown were entered simultaneously into the multivariable model. For categorical variables, the indicated reference category was used for comparison. The odds ratio for age represents the change in odds of dual sensory impairment associated with each one-year increase in age. We considered a two-sided p-value of <0.05 to be statistically significant. BMI, body mass index; CAD, coronary artery disease; COPD, chronic obstructive pulmonary disease

	OR	CI	P-value
Age	1.03	1.02-1.03	<0.001
BMI			
Healthy weight	Reference		
Underweight	1.24	0.83-1.84	0.13
Overweight	0.99	0.85-1.16	
Obese	1.15	0.97-1.35	
History of smoking			
None	Reference		
Yes	1.32	1.17-1.49	<0.001
Diabetes mellitus			
None	Reference		
Yes	1.11	0.95-1.29	0.20
Hypertension			
None	Reference		
Yes	1.21	1.01-1.37	0.004
Stroke			
None	Reference		
Yes	1.48	1.20-1.83	<0.001
CAD			
None	Reference		
Yes	1.34	1.11-1.61	0.002
Cancer			
None	Reference		
Yes	1.20	1.04-1.39	0.011
COPD			
None	Reference		
Yes	1.66	1.37-2.01	<0.001
Dementia			
None	Reference		
Yes	2.04	1.52-2.76	<0.001
Anxiety			
None	Reference		
Yes	1.69	1.45-1.98	<0.001
Depression			
None	Reference		
Yes	2.10	1.82-2.43	<0.001

Sensory impairment and functional outcomes

In this study, multivariate regression analysis revealed that all three impairments of interest (visual impairment alone, hearing impairment alone, or dual sensory impairment) were independently associated with impaired functioning across all assessed domains.

When ambulatory limitation was assessed, vision impairment alone and hearing impairment alone were found to have a twofold increase in the odds of impaired ambulation (aOR: 2.20 and aOR: 1.94, respectively; p < 0.001). Those with dual sensory impairment had the largest magnitude of impairment, with about a fourfold higher likelihood of impaired ambulation compared to participants without impairment (aOR: 3.87; p < 0.001) (Figure 1).

**Figure 1:**
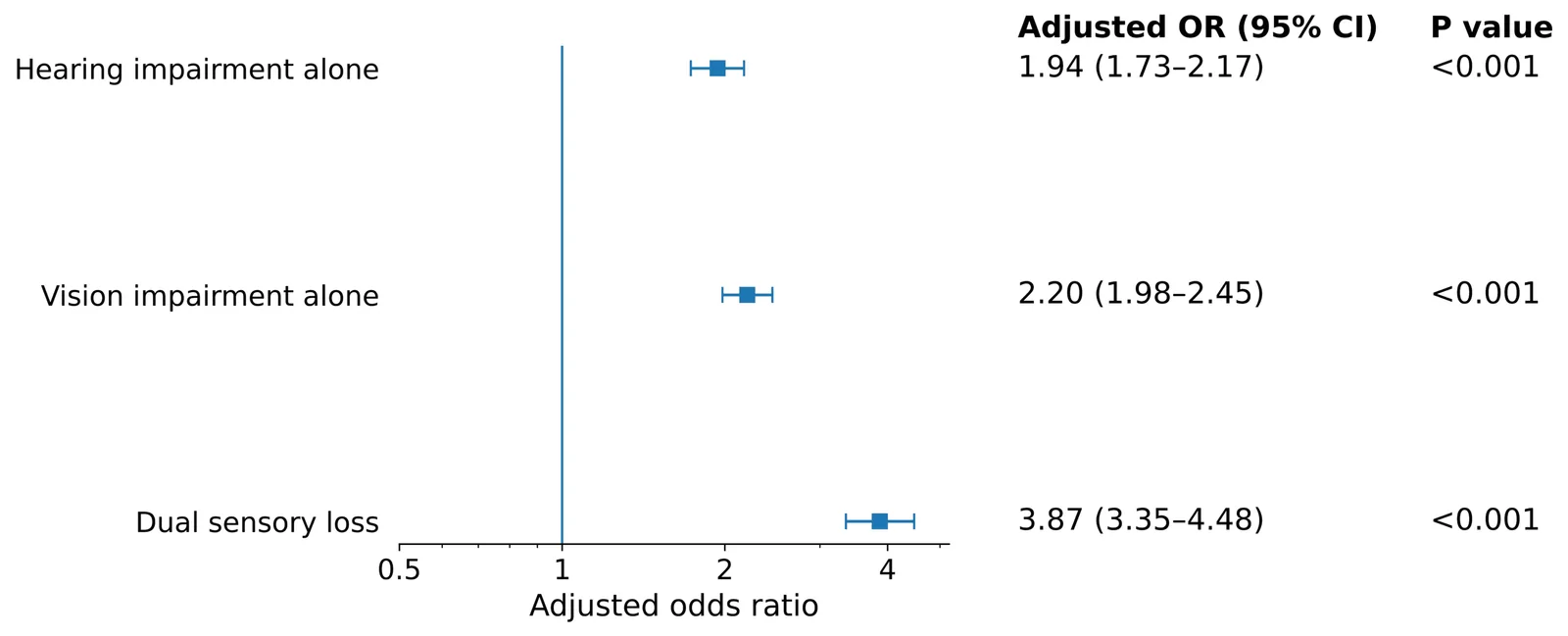
Factors associated with ambulatory limitation. Forest plot showing adjusted odds ratios (aORs) and 95% confidence intervals (CIs) for the associations of hearing impairment alone, vision impairment alone, and dual sensory loss with ambulatory limitation. Squares represent the aORs, and horizontal lines represent the corresponding 95% CIs. The vertical line at OR = 1 indicates the null association. Estimates were derived from multivariable logistic regression models accounting for the complex survey design, including sampling weights, stratification, and clustering by primary sampling unit.

Dual sensory impairment also had the largest magnitude of association with difficulty performing self-care (aOR: 3.55; p < 0.001), compared with visual impairment alone (aOR: 2.16) or hearing impairment alone (aOR: 1.62) (both p < 0.001) (Figure 2).

**Figure 2:**
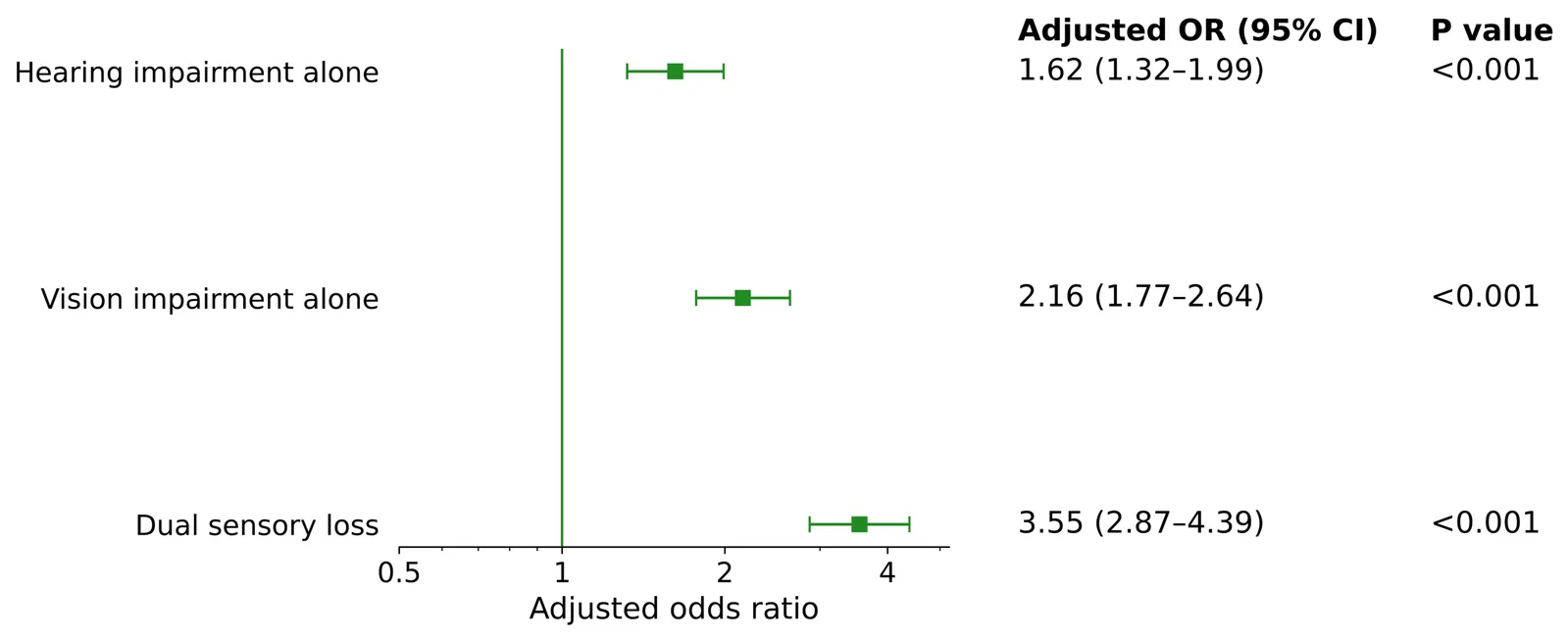
Factors associated with difficulty with self-care. Forest plot showing adjusted odds ratios (aORs) and 95% confidence intervals (CIs) for the associations of hearing impairment alone, vision impairment alone, and dual sensory loss with difficulty performing self-care activities. Squares represent the aORs, and horizontal lines represent the corresponding 95% CIs. The vertical line at OR = 1 indicates the null association. Analyses incorporated sampling weights, strata, and primary sampling unit clustering.

Difficulty communicating was another domain assessed in our analysis, as shown in Figure 3. Hearing impairment alone had a threefold increase in the likelihood of having difficulty communicating with others (aOR: 3.01; p < 0.001), whereas vision impairment alone had a lower but still substantial association with communication difficulties (aOR: 2.40; p < 0.001). Combined sensory impairment had a striking sixfold higher odds of having difficulty communicating with others (aOR: 6.03; p < 0.001).

**Figure 3:**
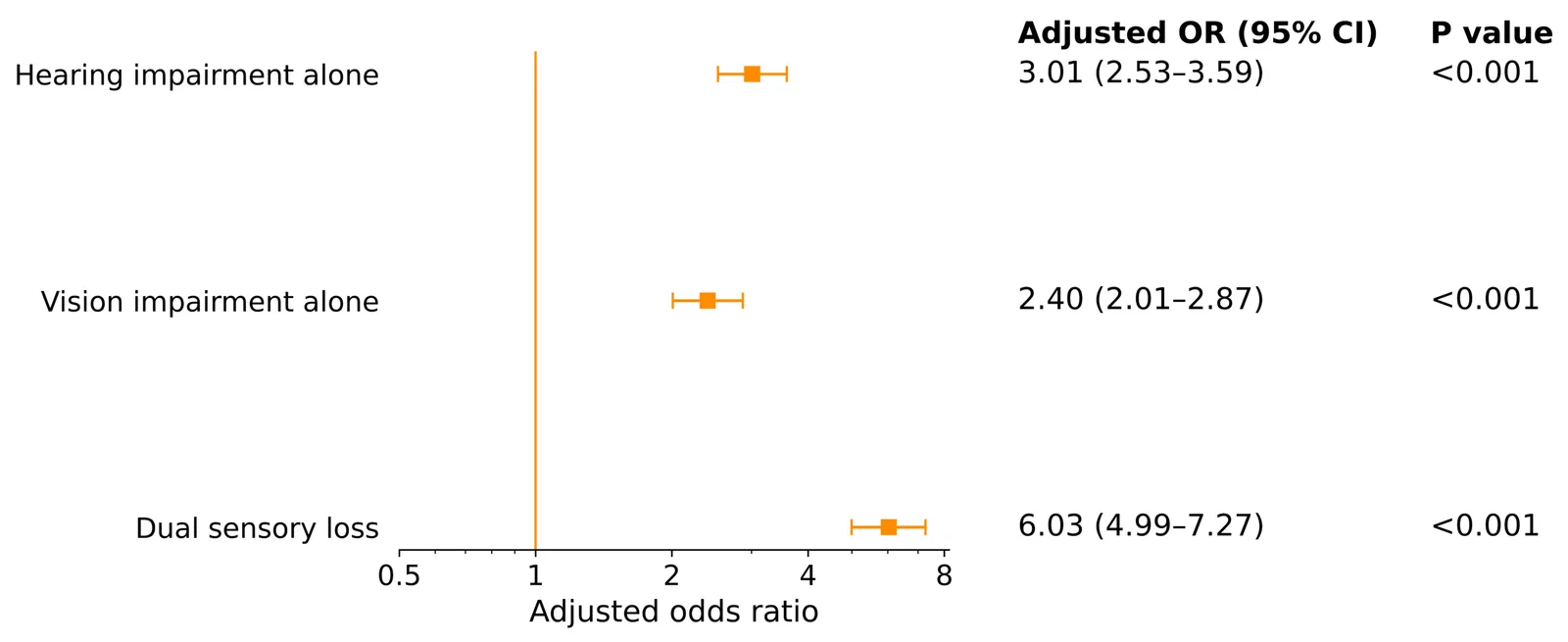
Factors associated with difficulty communicating. Forest plot displaying adjusted odds ratios (aORs) and 95% confidence intervals (CIs) for the associations of hearing impairment alone, vision impairment alone, and dual sensory loss with difficulty communicating. Squares indicate the point estimates, and horizontal lines indicate the 95% CIs. The vertical reference line at OR = 1 represents no association. Estimates were obtained from survey-weighted multivariable logistic regression models accounting for stratification and clustering.

As for difficulty performing errands independently (Figure 4), both hearing impairment alone and visual impairment alone had increased odds of having difficulty in performing errands independently (aOR: 1.74 and aOR: 2.31, respectively; both p < 0.001). Dual sensory impairment exceeded the observed effect seen in both sensory impairments alone (aOR: 3.81; p < 0.001).

**Figure 4:**
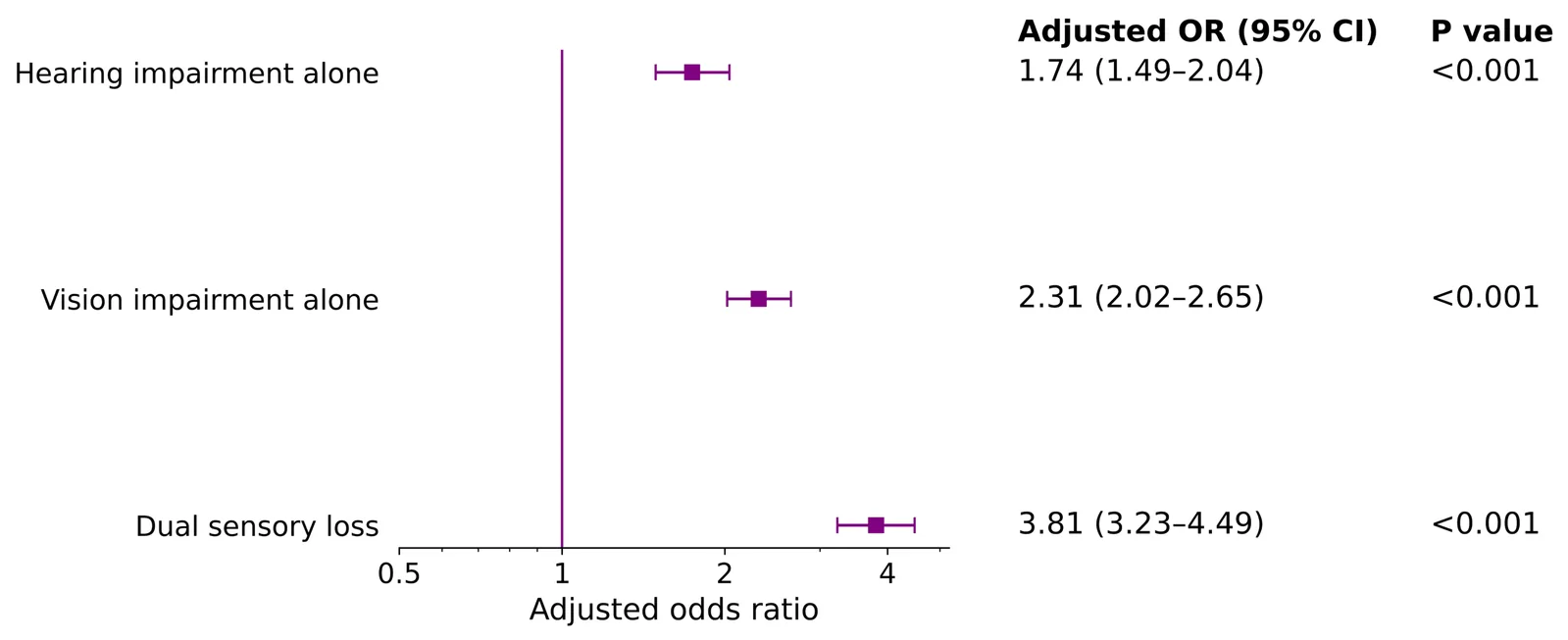
Factors associated with difficulty doing errands alone. Forest plot showing adjusted odds ratios (aORs) and 95% confidence intervals (CIs) for the associations of hearing impairment alone, vision impairment alone, and dual sensory loss with difficulty performing errands independently. Squares represent aORs, horizontal lines represent 95% CIs, and the vertical line at OR = 1 denotes the null association. The regression models accounted for survey weights, stratification, and clustering by primary sampling units.

Finally, dual sensory impairment showed the greatest magnitude of impairment in participation in social activities, with approximately fourfold higher odds of having difficulty (aOR: 4.08; p < 0.001). Hearing impairment alone and visual impairment alone were also independently associated with difficulties participating in social activities (aOR: 1.90 and aOR: 2.42, respectively; both p < 0.001) (Figure 5).

**Figure 5:**
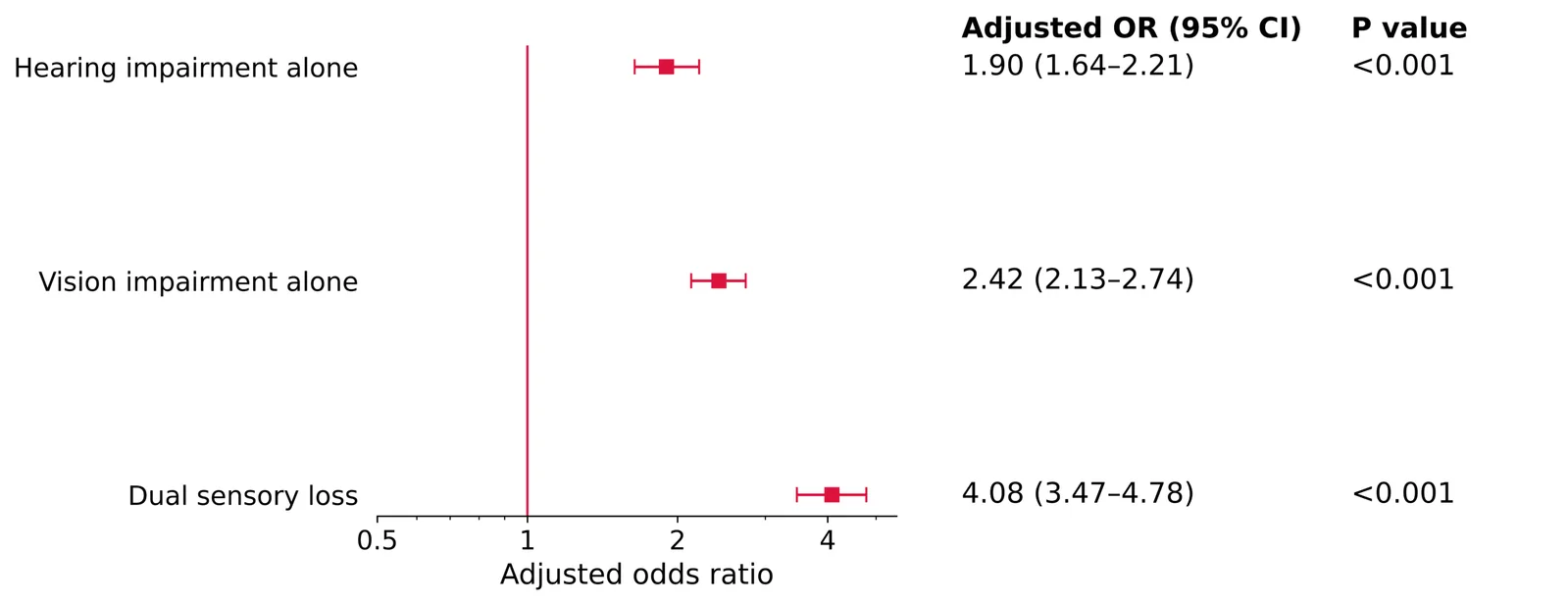
Factors associated with difficulty participating in social activities. Forest plot showing adjusted odds ratios (aORs) and 95% confidence intervals (CIs) for the associations of hearing impairment alone, vision impairment alone, and dual sensory loss with difficulty participating in social activities. Squares represent the aORs, and horizontal lines represent the corresponding 95% CIs. The vertical reference line at OR = 1 indicates no association. Estimates were obtained using multivariable logistic regression incorporating the complex survey design, including sampling weights, strata, and primary sampling units.

Across all domains, combined impairment showed consistent superiority, with the strongest association with functional impairment compared to isolated sensory impairments.

## Discussion

In our nationally representative study of the US population, dual sensory impairment was associated with poorer functional outcomes compared with isolated visual or hearing impairment. Combined sensory impairment was more prevalent among smokers, those with significant comorbid burden, and those with self-reported poorer health status and life satisfaction than among individuals without sensory impairment. These findings align with those previously described by Bright et al., highlighting that combined sensory impairment was associated with poorer quality of life and health status [5].

In our study, age showed an independent association with greater odds of dual sensory impairment. This finding aligns with evidence in the literature demonstrating that concurrent impairment in vision and hearing increases significantly with advancing age due to age-related ocular and auditory disorders, which frequently coexist in older individuals [5,6].

Individuals in our study with combined sensory deficits reported significantly poorer general health and life satisfaction than those without sensory impairments. These observations are consistent with previous studies described in the literature, as a systematic review evaluating the quality of life in older adults with sensory impairments concluded that sensory impairment in general was associated with lower quality of life, with the more severe the impairment, the lower the quality of life. Furthermore, it noted that individuals with dual sensory impairment had a worse quality of life compared to individuals with isolated sensory deficits [9].

Moreover, our analysis has concluded that several comorbidities were independently associated with combined sensory impairment after adjustment; these comorbidities are hypertension, stroke, coronary artery disease, chronic obstructive pulmonary disease, cancer, dementia, anxiety, and depression. This observation aligns with previously reported observations by Bright et al. [5]; it was shown that dual sensory impairment is linked to greater multimorbidity, poorer quality of life, increased mortality, and increased rates of depression compared to either isolated sensory impairment or no impairment at all [5,12]. Similarly, Schneider et al. described prominent associations between combined hearing and visual impairment and depression, cognitive decline, diminished independence, and reduced well-being in older adults [6].

Interestingly, diabetes mellitus was substantially more common among individuals with a combined sensory deficit but lost its association after multivariable adjustment. This suggests that confounding demographic or clinical covariates may explain the observed relationship in descriptive analysis rather than diabetes by itself.

Moreover, dual sensory impairment has shown the strongest independent association with impaired ambulation, self-care, communication, independence in performing errands, and social participation in comparison with visual or hearing impairment alone. Our findings align with those described in the literature, demonstrating that coexisting visual and hearing impairment imposes a larger functional burden compared with isolated sensory deficits alone [5,13,14].

Among the outcomes assessed, dual sensory impairment was strongly associated with impaired ability to ambulate, with a fourfold increase in limitation in ambulation among individuals with a combined sensory deficit. In Bright et al.’s study, it was reported that difficulty with mobility and increased disability were present among those with dual sensory impairment [5]. This conclusion closely aligns with our findings that the combined impairment of sensory inputs constitutes a larger association with ambulatory limitation compared to isolated sensory deficit.

Another functional domain explored in our analysis was difficulty with self-care. Dual sensory impairment was shown to be a strong independent factor associated with difficulty performing self-care. This might be because daily living requires continuous visual and auditory input; thus, an impairment of both sensory functions impairs an individual’s ability to carry out these tasks without assistance. Similar findings were reported in the literature, where adults with combined sensory deficits are more dependent in carrying out activities of daily living [15-18]. Therefore, the findings described in our analysis reinforce evidence from the literature demonstrating that dual sensory loss is associated with impaired personal independence.

Communication is also a functional domain explored in our analysis. It has been shown that communication had the largest relative effect in the study, with combined sensory impairment having sixfold higher odds of having difficulty in communication. This observation is expected as communication relies heavily on hearing and vision during interpersonal interactions. Vision helps in lip reading and the ability to recognize facial gestures and expressions and helps in environmental awareness, while hearing effectively contributes to speech perception. Previously published studies consistently demonstrated the association between dual sensory impairment and difficulty in communication. It has been reported that individuals with dual sensory impairment experience greater difficulty with communication, possibly contributing to other psychiatric comorbidities in this patient population [13,19].

Likewise, difficulty performing errands independently and participating in social activities were significantly associated with dual sensory impairment, as independent errands and socializing rely heavily on cognitive and sensory processing for environmental awareness, proper communication, and safe mobility. As demonstrated in the literature, individuals with dual sensory deficits have greater dependence on day-to-day activities and difficulty participating in social activities, in the form of difficulty shopping, mobilizing, participating in leisure activities, increased isolation, and reduced community participation [5,20].

This study has several strengths. Primarily, we used a large, nationally representative sample, which enhances the findings’ generalizability to the population of the United States. Our study examines visual, hearing, and dual impairment across several domains, which allows the direct evaluation of the relative impact. Also, multivariate regression analysis was utilized to adjust for potential demographic and clinical confounders, which reinforces the validity of the concluded associations. However, as this is a cross-sectional study, causality and temporality cannot be proven. Furthermore, the analyzed data were self-reported, which might be subject to recall bias or misclassification bias. Furthermore, residual confounding covariates from unmeasured factors cannot be excluded. Additionally, the status of vision and hearing impairment was self-reported and not validated by audiometric or ophthalmologic assessments, which may lead to the misclassification of impairment severity. Some caution is warranted in the interpretation of the particularly strong association observed between dual sensory impairment and communication difficulty. The hearing impairment exposure measure and the communication outcome measure are both based on related auditory processes, and there may be conceptual overlap between the two. Also, the gathered covariates for the multivariable models were selected based on literature-reported associations, but we cannot rule out that adjusting for some clinical or psychological comorbidities may be adjusting for some of the causal pathway between sensory impairment and functional decline for some participants, which could attenuate the observed associations. Finally, our sample was limited to non-institutionalized adults, and the findings may not be generalizable to institutionalized populations who often have a greater burden of sensory and functional impairment.

## Conclusions

In conclusion, dual hearing and visual impairment was found to be independently associated with poorer functioning across all included domains in our study compared with isolated sensory deficit. Furthermore, poorer self-reported health status and life satisfaction and a higher burden of chronic comorbidities were found to be more prevalent among those with dual sensory impairment. These observations demonstrate the importance of multidisciplinary and comprehensive patient management to improve the quality of life and maintain independence.
